# Developmental Psychology and Healthcare Professions: Autism Knowledge Among Nurses: An Observational Study

**DOI:** 10.3390/clinpract14060212

**Published:** 2024-12-12

**Authors:** Rosaria Ferrara, Giuseppe Campagna, Pasquale Ricci, Felice Damato, Lidia Ricci, Leonardo Iovino, Flavio Marti, Roberto Latina, Roberta Simeoli

**Affiliations:** 1 Department of Anatomy Histology, Legal Medicine and Orthopaedics, Sapienza University of Rome, Viale Regina Margherita 336, 00185 Rome, Italy; pasquale.ricci@uniroma1.it (P.R.); felicemarco.damato@unorima1.it (F.D.); lidia.ricci@uniroma1.it (L.R.); 2 Department of Medical-Surgical Sciences and of Translational Medicine, Faculty of Medicine and Psychology, Sapienza University of Rome, 00185 Rome, Italy; giuseppe.campagna@uniroma1.it; 3 Department of Economic and Legal Studies (DISEG), “Parthenope” University of Naples, 80133 Naples, Italy; leonardo.iovino@collaboratore.uniparthenope.it; 4 School of Nursing and Midwifery—S. Camillo, Faculty of Medicine and Psychology, Sapienza University of Rome, 00185 Rome, Italy; flavio.marti@uniroma1.it; 5 Department of Health Promotion Science, Maternal and Infant Care, Internal Medicine, and Medical Specialities (PROMISE), University of Palermo, 90128 Palermo, Italy; roberto.latina@unipa.it; 6 Department of Humanistic Studies, University of Naples Federico II, 80126 Naples, Italy; roberta.simeoli@unina.it; 7 Neapolisanit Research and Rehabilitation Center SRL, 80044 Ottaviano, Italy

**Keywords:** autism, developmental psychology, clinic psychology, social medicine, autism awareness, nursing

## Abstract

**Background:** One of the biggest limitations faced by autistic people is the lack of knowledge of their condition. Our study aims to evaluate and discuss the knowledge of autism among nurses, which is a social and health category often in close contact with autistic people. **Objective:** Given the limited exploration of awareness levels about autism among healthcare professionals, this study aims to investigate general and specific knowledge of autism within a group of nursing students enrolled in a master’s degree. **Methods:** A total of 66 nurses completed the questionnaire. Descriptive analyses were conducted on the results for the four subcomponents of the questionnaire: (i) general knowledge, (ii) symptomatology, (iii) screening and diagnosis, and (iv) intervention and treatment. A correlation analysis was performed between the participants’ demographic variables and questionnaire scores. Additionally, a multivariable logistic regression was conducted to analyze the association between the participants’ basic demographic characteristics and questionnaire scores. **Results:** Results showed a good percentage of correct answers in the “general knowledge” category. Furthermore, a good level of knowledge regarding the fact that ASD is a developmental disorder and a congenital disease also emerged. **Conclusions:** Regarding the knowledge of typical autism symptoms, participants answered most of the questions correctly. Correct answers decreased for questions related to screening and diagnosis. In particular, participants had limited knowledge of the DSM-5 and the timing of ASD diagnosis. Similar levels of knowledge were observed for the fourth category, “intervention and treatment”.

## 1. Introduction

Autism spectrum disorder (ASD) is a neurodevelopmental condition with childhood onset [1,2] that mainly involves difficulties in communication and social interactions and the presence of restricted and repetitive behaviors. Recent studies confirmed that ASD is also characterized by sensory peculiarities [3], inflammatory processes [4], and a wide spectrum of individual variations in cognitive functioning [5].

The Center for Disease Control and Prevention (CDC) has estimated that 1 in 44 children at the age of 8 are diagnosed with ASD, and the prevalence of the disorder is increasing compared to previous estimates [6]. A similar trend has been reported in Italy, where the prevalence of students with this disability has increased from 1.7% in the years 1989–1990 to 3.4% in the biennium 2017–2018 [7]. While epidemiological studies appear to indicate a significant increase in the phenomenon, several authors suggest that it may be only apparent and attributable to other factors, such as increased awareness of the disorder among families and healthcare professionals, the inclusion of cases within the spectrum that do not have intellectual disabilities or severe impairments, the refinement of diagnostic criteria, and advancements in early diagnosis.

On the other hand, despite the research efforts aimed at a better understanding of the disorder, it has only been a few years since knowledge of autism has begun to spread even among those who are not in the field (e.g., caregivers) [8].

In recent years, the scientific literature has been showing that the in-depth understanding of ASD characteristics is increasing, but many gaps are still present even for specialized clinicians [9].

However, several recent studies are shedding light on new conceptions of the disorder [9]. It is evident that a thorough knowledge of the autistic condition can facilitate early diagnosis and, more importantly, a more precise intervention, not only from a rehabilitative perspective but also in terms of comprehensive care [10,11,12].

Nevertheless, due to the phenotypic and etiological heterogeneity among individuals with ASD, early diagnosis of ASD remains a challenge [13,14]. Early intervention for children with ASD can improve their communication and social skills, as well as long-term prognosis and quality of life [15].

Although research is making progress in proposing alternative methods to promote early diagnosis [16], what still represents an obstacle is the awareness of the disorder. Early diagnosis depends not only on how specialized clinicians operate in the field but also on the awareness and knowledge of ASD by parents [17] and healthcare professionals who are not directly involved in the diagnostic process but may encounter a case of ASD and not recognize it promptly. These individuals could be crucial in alerting caregivers to the presence of suspicious signs and indicating the correct course of action [18,19].

Currently, the literature lacks assessments of the awareness levels regarding ASD among healthcare professionals. Some authors identified that nurses have inadequate knowledge about ASD screening practices, and this might be a major barrier to early identification and interventions [20,21,22].

In addition to the low levels of education on the subject, the literature highlights how nurses feel unprepared and unsafe and reports the great communication barrier that arises in interacting with ASD people [23]. For nurses, these issues are critical and lead to a series of failures to adapt to meet the needs of ASD patients. These adaptations refer to changes in the environment, organization, and procedures to allow people with disabilities to obtain adequate care. Changes in both the physical and social environment promote accessibility and equity and reduce anxiety and sensory stimuli [24,25].

Our aim was to measure the knowledge about autistic conditions among nurses in Italy. We aimed to shed light on this aspect among Italian healthcare professionals, focusing on nurses, just as was carried out in China by Ma [26].

Given the cross-disciplinary nature of their work, a nurse may easily encounter an individual with ASD, whether in pediatrics or any other department. Adequate awareness of the disorder and specific knowledge in this area could facilitate an early diagnostic process and enhance patient care by improving interactions and recognizing their needs, thereby reducing their communication efforts and associated frustration experience. This lack of knowledge is, in fact, one of the major barriers that individuals with ASD encounter when using healthcare services.

Although previous research has examined pediatric nurses’ experiences [27,28] and knowledge of ASD, it is still unclear what factors might predict their knowledge and understanding and associated correlates [29]. It is not known how well pediatric nurses understand and know autism spectrum disorders [30]. Researchers highlighted that during interactions, nurses experience mixed feelings, such as sadness, uneasiness, inadequacy, embarrassment, and tenderness. They declare the need for more knowledge about ASD. They would like to improve their own capacity for interactions with these children also through training [31,32]. These aspects indicate that there is a growing need for nurses and other healthcare professionals who have knowledge and skills in caring for children with ASD [33,34]. Thus, the aim of this study is to investigate the perceived knowledge of ASD among pediatric nurses working in Italian hospitals, specifically in relation to the four knowledge domains: (1) general knowledge of ASD, (2) the signs and symptoms of ASD, (3) the diagnosis and assessment of ASD, and (4) the intervention and treatment of ASD.

## 2. Methods

### 2.1. Investigation Site and Participants

The study was conducted in Rome, Italy, from December 2021 to March 2022. The survey subjects were 66 nurses in their first year of a master’s degree in nursing and already assigned to specific work settings. This was a convenience sample, which included all master’s degree nursing students from a university in central Italy.

### 2.2. Questionnaire Content

The questionnaire used was adapted from the study conducted by Ma et al. in 2021. The questions are based on the DSM-5 diagnostic criteria for ASD’s core symptoms, as well as the interventions indicated in the evidence-based ASD recommendations [1,33].

The questionnaire was divided into five sections: (i) the first section collected the participants’ basic demographic information, such as their age, gender, ethnicity, education, number of years in practice, occupation, professional title, and economic income; (ii) the second section included six questions designed to measure individuals’ general knowledge of ASD, such as whether ASD is a developmental disorder, a congenital disorder, or a hereditary disorder; (iii) the third section included seven questions about the knowledge of ASD symptomology, such as social disengagement or language problems; and (iv) the fourth section had five questions about ASD screening and diagnosis, including diagnostic criteria and age for ASD screening, and four questions were about knowledge of ASD intervention and treatment, including questions on behavioral interventions and medication therapy. All questions were multiple choice, with answers of “Yes”, “No”, and “I don’t know”. The instrument was then revised based on experts’ analysis and recommendations for face validity and content validity by a group of five researchers. The questions do not measure constructs but intend to probe whether or not one has certain knowledge.

### 2.3. Data and Statistical Analysis

The questionnaire consisted of 22 questions; one point was assigned for each correct answer, and no points were given for incorrect answers or for the response “I don’t know”.

Descriptive analysis was conducted for both the participants’ demographic characteristics and their awareness of ASD, grouped into the following subcomponents: (i) general knowledge, (ii) symptomatology, (iii) screening and diagnosis, and (iv) intervention and treatment. Categorical variables in Table 1, Table 2, Table 3, Table 4 and Table 5 are presented as absolute frequencies and percentages—n (%), while continuous variables in Table 6; Table 7 are shown as mean ± SD (standard deviation) and 95%CI (confidence interval).

## 3. Results

A total of 66 nurses participated in the study, and all completed the entire questionnaire. The basic demographic characteristics and professional information of the participants are shown in Table 1.

Participants were n = 48 (72.7%) females and n = 18 (27.2%) males with a mean age of 31.8 ± 8.6 (95%CI: 29.3 to 34.3) and 32.2 ± 6.9 (95%CI: 28.7 to 35.6), respectively (*p* = 0.3). Between the participants (female vs. male), there was no difference in education years: 14.4 ± 6.5 (95%CI: 12.5 to 16.3) vs. 13.4 ± 7.3 (95%CI: 9.8 to 17.1), *p* = 0.8.

Table 1 shows the descriptive demographic and professional information of the n = 66 participants.

A total of 54.5% of the nurses were under 30 years old, and 72.7% were females. Among the participants, 96.9% had no autistic relatives, and 58.2% of the operators affirmed that they had more than 5 years of work experience.

The normality of the continuous variables and of the residuals was verified by the Shapiro–Wilk test and checking the Q-Q plot. Homoscedasticity was verified by checking studentized residuals vs. fitted values plots.

Comparisons of variables with two levels/groups versus age, education years, general knowledge, symptomology, screening and diagnosis, intervention and treatment, and all question variables were evaluated by the Brunner–Munzel test (Table 6 and Table 7), while the comparisons of the continuous variables, in Table 6, with respect to variables with more levels were obtained by using a generalized linear mixed model with distribution = negative binomial. The choice of the negative binomial distribution allowed us to overcome the over-dispersion problem. In fact, the rate Pearson’s X^2^/DF (degrees of freedom) < 1.

The correlations between the variables in Table 8 were evaluated by Kendall’s *τ*_c_ because there were so many ties.

The cut-off (value = 17) of the total score was obtained by applying a mathematical procedure based on the ranking, more precisely using the “proc rank with ties = high” developed by SAS software. A value > 17 was considered the threshold for good knowledge of ASD in children.

The association between categorical variables and the score (17 < cut-off ≤ 17) shown in Table 7 was estimated by the X^2^ test.

Multivariable logistic regression was used to evaluate the association between the total score cut-off (dependent variable) and the independent variables shown in Table 9.

All analyses were performed by using SAS v. 9.4 TS Level 1M8 (SAS Institute Inc., Cary, NC, USA). A *p* < 0.05 was considered statistically detectable.

### 3.1. Participants’ Awareness and Knowledge of ASD in Children

The results of the questions in the “general knowledge” category show a percentage of correct answers exceeding 50% for as many as 6 out of 9 questions. Specifically, as indicated in Table 2, it is quite commonly known (78.7%) that the causes of ASD include genetic factors. There is also a good level of knowledge regarding the fact that ASD is a developmental disorder and a congenital disease, with correct response percentages of 51% and 54%, respectively. However, there is limited awareness about the life expectancy of individuals with ASD. In fact, 54.5% of the respondents believe that they have a shorter average lifespan compared to the non-autistic population.

Regarding the knowledge of typical autism symptoms, participants answered most of the questions correctly, reaching a mean percentage of 85% correct responses (Table 3).

The mean percentage of correct responses dropped to 65.7% for questions related to screening and diagnosis. Specifically, participants have little knowledge of the DSM-5 (36.3%) and the timing in which the diagnosis of ASD occurs (45.4%) (Table 4).

The mean percentage of correct answers for the fourth category, “intervention and treatment”, was 47.7%. Except for the question “ASD is incurable”, which showed good understanding, with 66.6% correct answers, for the other questions, the percentage of correct answers was less than 45%. In particular, knowledge regarding the drugs used for ASD and the types of treatments that are most commonly used and effective appears to be lower, with a percentage of 39.4%. (Table 5).

### 3.2. The Impact of Demographics on Questionnaire Responses

As evident from Table 6, none of the demographic or professional information variables had a significant influence on the questionnaire outcomes across all of its fourth components. In fact, all *p*-values were more than 0.05. These results were further confirmed by the analysis conducted considering the cut-off (value = 17) of the total score relative to all questions (Table 7). None of the variables appeared to be associated with the questionnaire outcome.

On the other hand, the correlation results have revealed a negative correlation between age and the score in the “intervention and treatment” category (Kendall’s *τ*_c_ = −0.20, *p* = 0.03) (Table 8).

In the multivariable logistic regression, none of the descriptive demographic and professional information of the participants was statistically associated with the total score cut-off of any question (≤17 and ≥17) (Table 9).

## 4. Discussion

Estimates of the prevalence of ASD in Italy have been increasing in recent years. In general, it is estimated that 1 child out of 77 between the ages of 7 and 9 receives an autism diagnosis, with a higher prevalence in males: males are 4.4 times more likely than females to receive an ASD diagnosis. This estimate has increased from 1.7% in the years 1989–1990 to 3.4% in the biennium 2017–2018 [7].

The aim of the present study was to investigate the knowledge among nurses regarding ASD, assuming that years of work and, therefore, experience could impact their knowledge. Furthermore, given that research on autism is constantly evolving, especially in terms of the diagnosis and individualization of specific symptoms, there are still ongoing scientific debates, and it is plausible to expect a lack of awareness on certain topics not only among non-professionals but also in the healthcare field.

Considering the extreme variability of ASD symptoms, another concern is the identification of the most appropriate treatment [35,36].

Moreover, delays in diagnosis are recorded, as well as significant difficulties during the diagnostic process, due to the absence of objective measures of the disorder and the fact that diagnosis relies solely on subjective measurements of behavior. The resulting delays in the process can have a significant impact on the lives of children with ASD, preventing them from receiving timely care and support [37].

Consistent with the described clinical scenario and with what is reported in the most recent literature [26], our results have highlighted gaps, especially in the areas of intervention and treatment, regarding which medical and rehabilitative treatments are most appropriate in relation to symptomatology and the DMS-5 diagnostic criteria. However, the overall level of knowledge is sufficient.

The results of our study appear to be in line with the current clinical situation and research on autism in Italy and worldwide. In fact, our study aligns with the recent literature, which increasingly emphasizes the importance of improving awareness of ASD among Italian healthcare professionals [34,38,39]. Moreover, there is limited awareness of the life expectancy of individuals with ASD. In fact, over 50% of the responses were incorrect. To improve the life expectancy of individuals with autism, there must be greater understanding, increased inclusion efforts, and more appropriate support and holistic care [40]. For this to occur, there needs to be increased collaboration between healthcare professionals and advocacy to fight stigma and enact policy changes. Nursing faculty’s ability to teach students about best practices in their care across the lifespan is important but not very common in academic education [41].

In Italy, for instance, [34] Corsano et al.’s 2020 study sheds light on the challenges healthcare professionals face in the clinical management of individuals with ASD. Nurses in their study expressed difficulties in their relationships and interactions with children. Specifically, they struggled to understand the children’s needs and to identify the best way to engage with them. Additionally, communication difficulties can hinder the early recognition of the specific needs of children with ASD, thus delaying healthcare intervention. As for Italian professionals, the knowledge gap abroad also appears to be primarily related to the areas of intervention and assessment, as demonstrated by other studies investigating nurses’ awareness of autism [20,21].

The results of this study show that no demographic variables have an impact on the questionnaire scores, meaning that years of work experience and education level do not influence the knowledge about autism. Such a small convenience sample is likely to reduce its statistical significance. These findings highlight that his level of knowledge could indeed reflect the general level of awareness about the disorder in Italy. As has been widely noted, research and clinical practice are generally struggling with some key issues related to autism, such as identifying the hallmark signs and personalizing treatment. Even the core symptoms of autism have been under scrutiny in recent years, with much research moving towards identifying objective measures that can support diagnosis [41,42]. Therefore, the lack of awareness in the healthcare sector regarding these issues, as confirmed by our study, represents a realistic snapshot of the clinical landscape of autism in Italy and the world. Further studies and in-depth exploration of autism are necessary, and the continuous education of healthcare professionals in this field is certainly essential to help them address this condition.

Ultimately, it is crucial for Italian healthcare professionals to gain a better awareness of ASD in order to ensure early and appropriate diagnosis and interventions for children with this condition. Further research and awareness initiatives can contribute to bridging this knowledge gap in Italy and improving the lives of individuals with ASD in the country.

## 5. Limitations

The limited size of our convenience sample may impact the generalizability of the study’s findings.

The study relied on a questionnaire that participants completed themselves, introducing the possibility of reporting bias and potentially leading to an overestimation of the findings related to ASD awareness. Our study was conducted considering only a limited geographic area within Italy, the capital city, Rome, without a rigorous validation of the instrument. As a result, the findings only reflect the situation in the capital city, where many young students converge from various regions for their professional training. To obtain a more comprehensive understanding of ASD awareness and knowledge levels in Italy, further large-scale, multicenter studies are required.

## 6. Conclusions

We conducted a questionnaire-based study to assess the levels of awareness and knowledge of autism among Italian healthcare professionals, particularly nurses, involved in various work settings that may not have direct involvement with ASD specifically but could potentially encounter cases and play a significant role in early symptom detection.

Our findings indicate a generally sufficient overall knowledge of autism, but some areas are lacking, particularly those related to diagnosis and intervention. These results reflect the current clinical and research landscape of autism and the existing gaps in knowledge in the field. Therefore, further studies to deeply understand the disorder are necessary in terms of timely and effective interventions, symptomatology definition, and the diagnostic process.

## Figures and Tables

**Table 1:** Descriptive demographic and professional information of the n = 66 participants.

Parameter	n (%)
Age (years)	
<30	36 (54.5)
30 to 40	20 (30.3)
40 to 50	4 (6.1)
≥50	6 (9.1)
Sex	
Female	48 (72.7)
Male	18 (27.2)
University degree	
Bachelor’s	10 (15.1)
Master’s	56 (84.8)
Autistic relative	
No	64 (96.9)
Yes	2 (3.03)
Working experience	
No	11 (16.6)
Yes	55 (83.3)
Years of work	
0 to 3	11 (20.0)
3 to 5	12 (21.8)
≥5	32 (58.2)
Work with autistic patients	
No	41 (74.5)
Yes	14 (25.4)

**Table 2:** Questions on general knowledge.

Parameter	n (%)
International Autism Day is on 2 April every year	
Correct	27 (40.9)
Incorrect	7 (10.6)
Not sure	32 (48.4)
ASD is a common disease	
Correct	24 (36.3)
Incorrect	33 (50.0)
Not sure	9 (13.6)
ASD is a developmental disorder	
Correct	34 (51.5)
Incorrect	26 (39.3)
Not sure	6 (9.1)
ASD is a congenital disease	
Correct	36 (54.5)
Incorrect	17 (25.7)
Not sure	13 (19.7)
The causes of ASD include genetic factors	
Correct	52 (78.7)
Incorrect	0 (0.0)
Not sure	14 (21.2)
There are state subsidies for families with ASD	
Correct	42 (63.6)
Incorrect	2 (3.0)
Not sure	22 (33.3)
The mean age life expectancy of an autistic is the same as that of the non-autistic population	
Correct	10 (15.1)
Incorrect	36 (54.5)
Not sure	20 (30.3)
There are regularly recruited autistic adults working	
Correct	55 (83.3)
Incorrect	0 (0.0)
Not sure	11 (16.6)
Sensory perception in autistic people is the same as in the norm typical population	
Correct	41 (62.1)
Incorrect	10 (15.1)
Not sure	15 (22.7)

**Table 3:** Questions on the symptomology.

Parameter	n (%)
The clinical manifestations of ASD can be mild or severe	
Correct	60 (90.9)
Incorrect	2 (3.0)
Not sure	4 (6.1)
Children with ASD may appear to have special skills or interests in a particular aspect	
Correct	62 (93.9)
Incorrect	1 (1.5)
Not sure	3 (4.5)
The IQ scores of children with ASD are either partially high or low or normal compared to those in the general population	
Correct	58 (87.8)
Incorrect	2 (3.0)
Not sure	6 (9.1)
Children with ASD may be unable to speak at an age when they should be able to	
Correct	59 (89.3)
Incorrect	0 (0.0)
Not sure	7 (10.6)
Children with ASD are indifferent to their surroundings, play alone and exhibit social withdrawal	
Correct	51 (77.2)
Incorrect	9 (13.6)
Not sure	6 (9.1)
Children with ASD do not respond to being called names	
Correct	46 (69.7)
Incorrect	3 (4.5)
Not sure	17 (25.7)
Children with ASD cannot use their own expressions of emotion to get your attention	
Correct	59 (89.3)
Incorrect	1 (1.5)
Not sure	6 (9.1)

**Table 4:** Questions on screening and diagnosis of ASD in children.

Parameter	n (%)
Children with ASD should go to medical institutions for consultation and treatment	
Correct	51 (77.2)
Incorrect	3 (4.5)
Not sure	12 (18.1)
A clinic for children with ASD includes a children’s neurology department, children’s health department and other departments	
Correct	58 (87.8)
Incorrect	0 (0.0)
Not sure	8 (12.1)
The DSM-5 has the latest diagnostic criteria for ASD	
Correct	24 (36.3)
Incorrect	2 (3.0)
Not sure	40 (60.6)
Early detection and early screening are helpful to the prognosis of ASD	
Correct	54 (81.8)
Incorrect	4 (6.1)
Not sure	8 (12.1)
The earliest age for the early screening of ASD is one year old	
Correct	30 (45.4)
Incorrect	11 (16.6)
Not sure	25 (37.8)

**Table 5:** Questions on the intervention and treatment of ASD in children.

Parameter	n (%)
There are no effective drugs to treat ASD	
Correct	26 (39.3)
Incorrect	20 (30.3)
Not sure	20 (30.3)
Behavioral interventions for ASD include applied behavioral analysis therapy, picture vocabulary communication systems	
Correct	26 (39.3)
Incorrect	0 (0.0)
Not sure	40 (60.6)
ASD is incurable	
Correct	44 (66.6)
Incorrect	18 (27.2)
Not sure	4 (6.0)
Vitamin supplements during pregnancy may prevent ASD	
Correct	30 (45.4)
Incorrect	5 (7.5)
Not sure	31 (46.9)

**Table 6:** Association of the basic demographic characteristics of the participants with questionnaire score.

Parameter	General Knowledge Mean ± SD; (95%CI)	Symptomology Mean ± SD; (95%CI)	Screening/Diagnosis Mean ± SD; (95%CI)	Intervention/Treatment Mean ± SD; (95%CI)	All Questions Mean ± SD; (95%CI)
Age					
<30	3.4 ± 1.2; (3.0 to 3.8)	5.8 ± 1.2; (5.4 to 6.2)	3.3 ± 1.0; (2.9 to 3.6)	2.1 ± 1.0; (1.7 to 2.4)	16.4 ± 3.6; (15.2 to 17.7)
30 to 40	2.7 ± 1.3; (2.1 to 3.3)	5.9 ± 1.1; (5.4 to 6.4)	2.8 ± 0.8; (2.3 to 3.2)	1.7 ± 1.2; (1.2 to 2.3)	14.5 ± 3.9; (12.6 to 16.3)
40 to 50	4.5 ± 0.5; (3.5 to 5.4)	7.0 ± 0.0; (n.e. to n.e.)	4.2 ± 0.9; (2.7 to 5.7)	1.5 ± 0.5; (0.5 to 2.4)	19.2 ± 1.5; (16.8 to 21.6)
≥50	3.1 ± 1.1; (1.9 to 4.3)	6.5 ± 0.5; (5.9 to 7.1)	4.2 ± 0.4; (3.7 to 4.6)	1.5 ± 1.1; (0.4 to 2.6)	16.5 ± 1.8; (14.5 to 18.4)
*p*	0.2	0.7	0.2	0.6	0.1
Sex					
Female	3.2 ± 1.3; (2.8 to 3.6)	6.1 ± 1.1; (5.7 to 6.4)	3.3 ± 1.0; (3.0 to 3.6)	1.9 ± 1.1; (1.5 to 2.2)	16.2 ± 3.3; (15.2 to 17.1)
Male	3.3 ± 1.2; (2.7 to 3.9)	5.6 ± 1.2; (5.0 to 6.3)	3.2 ± 1.0; (2.7 to 3.7)	1.9 ± 1.1; (1.3 to 2.5)	15.6 ± 4.5; (13.3 to 17.8)
*p*	0.84	0.52	0.86	0.91	0.4
University degree					
Bachelor’s	2.6 ± 1.4; (1.5 to 3.6)	5.9 ± 1.2; (5.0 to 6.7)	3.5 ± 0.9; (2.8 to 4.2)	1.4 ± 0.7; (0.9 to 1.9)	14.6 ± 4.0; (11.7 to 17.5)
Master’s	3.3 ± 1.2; (3.0 to 3.7)	6.0 ± 1.2; (5.6 to 6.3)	3.2 ± 1.0; (2.9 to 3.5)	2.0 ± 1.1; (1.7 to 2.3)	16.3 ± 3.6; (15.3 to 17.2)
*p*	0.2	0.9	0.6	0.2	0.1
Autistic relative					
No	3.2 ± 1.2; (2.9 to 3.5)	5.9 ± 1.2; (5.6 to 6.2)	3.2 ± 1.0; (3.0 to 3.5)	1.9 ± 1.1; (1.6 to 2.1)	15.9 ± 3.7; (15.1 to 16.9)
Yes	4.0 ± 1.4; (−8.7 to 16.7)	6.5 ± 0.7; (0.1 to 12.8)	4.0 ± 1.4; (−8.7 to 16.7)	2.0 ± 1.4; (−10.7 to 14.7)	18.5 ± 2.1; (−0.5 to 37.5)
*p*	0.5	0.9	0.5	0.2	0.3
Working experience					
No	3.1 ± 1.3; (2.2 to 4.0)	6.2 ± 0.9; (5.5 to 6.8)	3.6 ± 0.9; (3.0 to 4.2)	2.2 ± 1.3; (1.2 to 3.1)	16.7 ± 4.3; (13.7 to 19.6)
Yes	3.2 ± 1.2; (2.9 to 3.6)	5.9 ± 1.2; (5.6 to 6.2)	3.2 ± 1.0; (2.9 to 3.5)	1.8 ± 1.0; (1.5 to 2.1)	15.9 ± 3.5; (14.9 to 16.8)
*p*	0.6	0.7	0.4	0.4	0.6
Years of work					
0 to 3	15.2 ± 2.9; (13.2 to 17.1)	6.0 ± 1.2; (5.1 to 6.8)	3.5 ± 0.6; (3.1 to 4.0)	2.1 ± 0.7; (1.6 to 2.5)	16.6 ± 2.9; (14.6 to 18.6)
3 to 5	14.5 ± 2.8; (12.7 to 16.4)	5.6 ± 1.1; (4.9 to 6.3)	3.1 ± 1.1; (2.3 to 3.7)	2.1 ± 1.2; (1.3 to 2.8)	16.5 ± 3.0; (14.5 to 18.4)
≥5	13.9 ± 3.5; (12.6 to 15.2)	6.0 ± 1.2; (5.5 to 6.4)	3.2 ± 1.1; (2.7 to 3.5)	1.6 ± 1.0; (1.3 to 2.1)	15.4 ± 3.9; (14.0 to 16.8)
*p*	0.4	0.9	0.7	0.4	0.5
Work with autistic patients					
No	3.1 ± 1.3; (2.7 to 3.5)	6.0 ± 1.2; (5.6 to 6.3)	3.1 ± 1.0; (2.8 to 3.5)	1.8 ± 1.1; (1.5 to 2.2)	15.7 ± 3.7; (14.6 to 16.9)
Yes	3.7 ± 1.1; (3.1 to 4.3)	5.7 ± 1.2; (4.9 to 6.4)	3.3 ± 1.0; (2.7 to 3.9)	1.8 ± 0.6; (1.4 to 2.2)	16.2 ± 3.1; (14.4 to 18.1)
*p*	0.3	0.6	0.6	0.8	0.5

Total score was calculated as the sum of the scores of each question. It assigned 1 to a “correct” response and 0 to “incorrect” or “not sure” responses. Abbreviation: (95%CI) = 95% confidence interval.

**Table 7:** Association between demographic and professional information of the participants versus total score cut-off of all questions.

Parameter	All Questions		
	≤17 (n = 44) n (%)	>17 (n = 22) n (%)	*p*
Age * (years)	31.8 ± 8.2; (29.3 to 34.3)	32.1 ± 8.2; (28.4 to 35.7)	0.9
Education years * (years)	13.6 ± 6.9; (11.5 to 15.7)	15.2 ± 6.4; (12.3 to 18.0)	0.3
Age (years)			0.31
<30	24 (66.6)	12 (33.3)	
30 to 40	14 (70.0)	6 (30.0)	
40 to 50	1 (25.0)	3 (75.0)	
≥50	5 (83.3)	1 (16.6)	
Sex			0.56
Female	33 (68.7)	15 (31.2)	
Male	11 (61.1)	7 (38.8)	
University degree			0.4
Bachelor’s	8 (80.0)	2 (20.0)	
Master’s	36 (64.2)	20 (35.7)	
Autistic relative			0.9
No	43 (67.2)	21 (32.8)	
Yes	1 (50.0)	1 (50.0)	
Working experience			0.7
No	8 (72.7)	3 (27.2)	
Yes	36 (65.4)	19 (34.5)	
Work with autistic patients			0.5
No	28 (68.2)	13 (31.7)	
Yes	8 (57.1)	6 (42.8)	

* Data are presented as mean ± SD; (95%CI): (95% confidence interval).

**Table 8:** Correlation between demographic and professional information of the participants with their knowledge scores on the characteristics of ASD in children.

Parameter	General Knowledge Kendall’s *τ*_c_; (95%CI)	Symptomology Kendall’s *τ*_c_; (95%CI)	Screening/Diagnosis Kendall’s *τ*_c_; (95%CI)	Intervention/Treatment Kendall’s *τ*_c_; (95%CI)	All Questions Kendall’s *τ*_c_; (95%CI)
Age (years)	−0.1; (−0.3 to 0.1) *p* = 0.2	0.1; (−0.03 to 0.3) *p* = 0.1	0.02; (−0.2 to 0.2) *p* = 0.8	−0.2; (−0.3 to −0.04) *p* = 0.03	−0.07; (−0.2 to 0.1) *p* = 0.4
Education years (years)	0.05; (−0.2 to 0.2) *p* = 0.5	−0.2; (−0.3 to 0.03) *p* = 0.07	−0.04; (−0.2 to 0.2) *p* = 0.6	-0.03; (−0.2 to 0.2) *p* = 0.7	−0.02; (−0.2 to 0.2) *p* = 0.8

Abbreviation: (95%CI) = 95% confidence interval.

**Table 9:** Multivariable logistic regression of demographic and professional information of the participants with respect to total score cut-off of all questions (≤17 and ≥17).

Parameter	OR	(IC95%)	*p*-Value
Age * (years)	1.01	(0.9 to 1.1)	0.8
Education years	1.06	(0.9 to 1.2)	0.3
Sex			
Male vs. Female	0.6	(0.1 to 2.7)	0.5
University degree			
Bachelor’s vs. Master’s	0.6	(0.1 to 4.3)	0.6
Autistic relative			
Yes vs. No	1.3	(0.06 to 31.3)	0.8
Years of work			
0–3 vs. 5	1.2	(0.2 to 7.7)	0.2
3–5 vs. 5	0.7	(0.1 to 4.6)	0.7
Work with autistic patients			
Yes vs. No	1.6	(0.4 to 6.2)	0.4

* Data are presented as mean ± SD.

## Data Availability

The data are not publicly available due to privacy. Data used in the present study are available from the corresponding author upon reasonable request.
